# Students’ Perspectives on Digital Psychotherapy—Possible Solutions for Digital Inpatient-Like Care Concepts: Qualitative Interview Study

**DOI:** 10.2196/82830

**Published:** 2026-06-01

**Authors:** Rebekka Robitzsch, Tania Lalgi, Alexander Diel, Sophie Schulz genannt Menningmann, Lucy Ann Gresser, Patrick Jonas Wollenberg, Martin Teufel, Alexander Bäuerle, Anita Robitzsch

**Affiliations:** 1 Clinic for Psychosomatic Medicine and Psychotherapy, LVR-University Hospital, University of Duisburg-Essen, Virchowstr, 174, Essen, 45147, Germany, +49 201 43 87 55 203; 2 Center for Translational Neuro- and Behavioral Sciences (C-TNBS), University of Duisburg-Essen, Essen, Germany

**Keywords:** e-mental health, digital mental health, artificial intelligence, AI psychotherapy, mental health care, psychiatric care, acceptance

## Abstract

**Background:**

The demand for mental health treatment is increasing, while the availability of treatment remains insufficient to meet the rising demand. Alternative solutions need to be explored to enable access to care for patients who cannot participate in traditional psychotherapeutic settings due to common barriers like place of residence, professional obligations, or physical limitations.

**Objective:**

This study aimed to investigate attitudes toward digital psychotherapy, specifically within a digital inpatient-like therapy setting, among psychology and medical students. These students represent the future generation of therapists and possess the educational background necessary to develop innovative ideas to benefit a digital psychotherapeutic setting.

**Methods:**

We conducted qualitative, semistructured interviews with 20 participants (10 psychology students and 10 medical students). The data were analyzed using an inductive, thematic analysis according to the methodology outlined by Braun and Clarke.

**Results:**

The thematic analysis led to a codebook including 4 overarching categories: (1) evolution of digitalization in medical practice, (2) future directions for digital psychotherapy, (3) technical framework, and (4) artificial intelligence–based psychotherapy.

**Conclusions:**

In the context of mental health, digital psychotherapy is accepted as a viable option when conventional face-to-face therapy is not possible. The primary concerns were potential impairments in the therapeutic relationship and interaction. Artificial intelligence was rejected as a standalone therapy but was considered acceptable as a supplementary tool. Technical problems represent a major obstacle for the consistent and reliable implementation of digital psychotherapy. A successful digital psychotherapeutic concept for inpatient and outpatient settings needs to enable a sufficient interpersonal therapeutic relationship situated within a reliable technical framework.

## Introduction

Mental illnesses are among the most common disorders worldwide. Current data indicate that over 12% of the global population lives with a mental health disorder [1]. Regarding the German population, a report from the Robert-Koch Institute highlights the increasing prevalence of depressive and anxiety symptoms in recent years [2]. Consistent with these findings, the German federal chamber of psychotherapists states that the need for psychotherapeutic treatment has increased in the last 2 decades [3]. However, patients endure waiting time for outpatient therapy from weeks to several months [4-6]. Severe cases or those with certain circumstances necessitate inpatient care [7-9]. From an economic perspective, inpatient care is considerably more expensive. A report from the German Psychotherapists Association estimated the 2019 costs of inpatient mental health care at €25 billion (US $29.4 billion), making it the most expensive specialty in the inpatient treatment sector in Germany [10]. In contrast, the costs for outpatient psychotherapy are significantly lower, at around €2.7 billion (US $3.2 billion) annually [10].

In response to the urgent need for psychotherapeutic treatment while simultaneously aiming to reduce costs for the health care system, the concept of inpatient-equivalent treatment (Stationsäquivalente Behandlung [StäB]) emerges as a realistic solution. This treatment model was implemented in 2018 in Germany [11]. StäB aims to enable mental health treatment by a multidisciplinary team that provides care at the patient’s home, which is equivalent to an inpatient treatment in a hospital [12]. Initial results from specialties like psychiatry [13], geriatric psychiatry [14], and child and adolescent psychiatry [15] appear promising. These studies report positive effects on quality of life [14], mental health status, involvement of relatives, efficacy, acceptance of treatment [15], and a reduction in inpatient readmissions [13]. This leads to the conclusion that the StäB concept could be a viable solution to all therapeutic fields. Schwarz et al [16] suggest that utilization of the StäB therapeutic model could be facilitated through digital mental health offerings. This combination could be a promising solution to the sector in general.

Rojas et al [17] suggest that internet-based interventions and digital technologies could help in addressing the shortage and unequal distribution of psychotherapy treatments. Digital options for the treatment of mental health issues were increasingly established during the COVID-19 pandemic because they could be used regardless of time or location [18]. In principle, the regulatory and financial framework in Germany is already set by the Digital Supply Act (Digitale-Versorgungs-Gesetz), which legally enables payment for certain digital health therapeutics by German health insurances [19]. Numerous studies have demonstrated positive effects of digital mental health interventions [20-24], even being supportive to inpatient treatment settings [25,26]. The umbrella term “digital interventions” incorporates a variety of therapeutic offerings delivered through digital media, and these may be accompanied by a therapist to varying degrees but also be available for self-help [27].

The related studies confirm that remotely delivered psychotherapy is accessible, manageable, and cost-effective [28,29], but there is less evidence for video-based psychotherapy than for app-based interventions delivered through a smartphone or the web [30]. Nevertheless, there is some support for video-based therapy [31], with some even calling it comparable [28,32]. Some authors go a step further and ask for the implementation of video-based therapy and digital interventions as an additional support [33], for example, apps, web-based offerings, group therapy, and occasional psychiatric consultations [28].

In response to the need for innovative approaches to digital psychotherapy settings, the aim of this study was to investigate the perspectives of medical and psychology students on digital psychotherapy and their opinions on the feasibility of our digital inpatient-like psychotherapy (DIPT) concept. The median age of German students in the last 3 semesters was a few months over 23 years [34], which makes most of them so-called “digital natives,” defined as a generation that has used digital media throughout their entire lives [35]. This characteristic could lead to a more intuitive, unprejudiced perspective on the application of digital media and allow them to better see the full potential of digital media compared to older generations. Braun et al [36] recently conducted qualitative interviews with psychology and medical students, investigating their information needs and preferences on digital mental health services. They aimed to consider the perspective of students as future therapists [36].

This particular approach in which the authors focus on students’ opinions remains underrepresented in psychotherapy research. Until now, students have often been investigated as the end users of specific interventions such as digital questionnaires [37], online lessons [38], or digital mental health programs [39]. Moreover, there are also studies investigating the effects of digital interventions in general [22,23,40], which include several digital approaches at once. This discrepancy is concerning when considering the unique value of the students’ perspective. As young, sometimes self-affected individuals who are well educated in relevant fields, they can provide a holistic perspective on the research question as potential future therapists. Recent findings indicate an open attitude toward the use of e-mental health apps [41].

**Figure 1.**
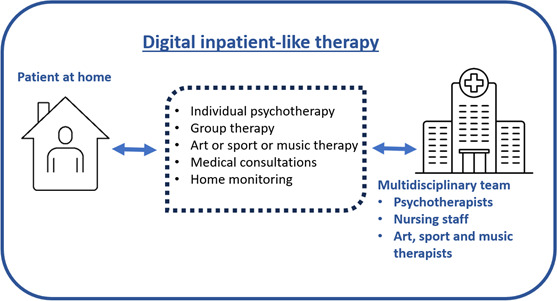
The digital inpatient-like therapy concept.

In this study, semistructured interviews were conducted to capture these unique perspectives regarding possible digital solutions addressing the current unmet needs in psychotherapeutic treatment in Germany. Specifically, the study explored the perspectives of psychology and medical students on a hypothetical DIPT concept. We envisioned a clinic in a virtual environment, where a digital application on an electronic device would provide access to all day-clinic services. All therapeutic modalities should be provided digitally, with representation from all relevant professional disciplines. The preferences for the specific design of such a therapeutic concept were explored in the interviews. Figure 1 provides a schematic overview. Based on these insights, attitudes were examined, along with expected opportunities and barriers. Additionally, we examined attitudes toward the use of artificial intelligence (AI) in psychotherapy, which is emerging as the next advancement in the field [42,43]. Gaining a deeper understanding of these aspects contributes to the development of realistic, patient-centered approaches to digital psychotherapy and also to the investigation of broader digital treatment pathways.

## Methods

### Study Design

To address our research objectives, we employed a qualitative interview study design to focus on the lived experiences and perspectives of each respondent [44]. This study method is an effective strategy for developing theories and exploring experiences of social situations and reasons for behavior [45].

### Ethical Considerations

Initially, ethical approval was obtained from the Ethics Committee of the Medical Faculty of the University of Duisburg-Essen (23‐11642-BO). The study was conducted after obtaining informed consent from all participants. Privacy of the participants was safeguarded by pseudonymizing each interview. The audio data were stored on a password-protected device and will be kept for the next 10 years, ensuring adequate confidentiality. Participation was voluntary, and the study participants did not receive any compensation.

### Recruitment and Participants

A total of 20 interviews were conducted between March and May 2024, involving 10 students from each of the fields of interest: medicine and psychology. The inclusion criteria were fluency in the German language, adult age (>18 y), and therapeutic-related study subject (human medicine or psychology). Participants were recruited in different ways, including flyers distributed at the university campus, in the clinic setting, on social media, and through direct communication, both verbally and through email, within the study team’s environment (professional and personal networks). Every interviewee completed the whole interview.

### Semistructured Interview

The majority of qualitative interview formats are semistructured interviews conducted with individuals or groups [46]. A semistructured interview comprises a guide of questions but allows for an individual order of questions, wording, and in-depth inquiry [45]. After reviewing the current relevant literature, we developed an interview guide adapted to our target group. The semistructured questionnaire is provided in Table S3 in Multimedia Appendix 1. Initial pilot testing ensured familiarity with the equipment and the interview process.

The interview was divided into 4 thematic sections. It started with questions about individual sociodemographic data and general and health-related media use. After that, we explored participants’ perspectives regarding our DIPT concept. The fourth section addressed experiences and expectations regarding digitalization in medical and psychotherapeutic contexts, including the possibility of AI-guided psychotherapy. Finally, we concluded by offering participants the opportunity to ask questions about the study or provide feedback. The interview guide, which provides a detailed definition of the DIPT concept, is provided in Table S3 in Multimedia Appendix 1.

### Data Collection

After a detailed description of the study was provided verbally and in written form, written informed consent was obtained and documented with signature. Interviews were conducted in person at the facilities of the LVR-University Hospital Essen, Clinic for Psychosomatic Medicine and Psychotherapy, Essen, Germany. Audio recordings were obtained with a voice recorder (Sony-ICD-PX470). We selected a sample of 20 interviews, with each subgroup consisting of 10 interviews, because recent studies indicate a saturation of themes with no more aspects emerging at this point [47,48]. Saturation of comprehension requires approximately 20 interviews [48], which was also achieved in this study when subgroups were analyzed separately. The interview guide was successfully applied. Therefore, all interviews yielded 20 comparable and complete datasets. Thereafter, audio data were transformed into written transcripts using f4x 2024 software [49]. Finally, the data underwent a proofreading process and were carefully collated with the audio recordings to ensure high data quality.

### Data Analysis and Quality Control

The average duration of an interview was approximately 29 minutes. The conducted interviews were transformed into pseudonymized transcripts. Data were analyzed semantically using MAXQDA 24 software, a tool that can be used for analyzing qualitative data [50,51]. Three independent researchers analyzed the data: 2 of them coding all transcripts, and the third coder coding 25% of the transcripts. The third researcher also acted as a supervisor and mediated in cases of disagreements, as described by Kuckartz [52].

We conducted a thematic analysis following the instructions established and revised by Braun and Clarke [53,54]. This approach recognizes the active role of the researchers in identifying and interpreting patterns of meaning across the data. The 6-phase framework consists of (1) familiarizing oneself with the data, (2) initial coding, (3) searching for themes, (4) reviewing themes, (5) determining themes, and (6) reporting the results. Themes were generated inductively through an iterative and reflective process, which was data-driven without a preestablished, potentially restrictive codebook [53]. The subjectivity and positionality of the research team (consisting of PhD candidates, research assistants, postdoctoral researchers, and a full-time professor) are considered integral to the analytic process. Thematic analysis is described as a 3-step approach of descriptive coding, interpretive coding, and the final definition of relevant themes [55]. After every step of this analysis, we held a meeting of the 3 coders to ensure the validity of the results [56]. The underlying methodological perspective was a coding reliability approach within reflexive thematic analysis, one of the several subtypes that have evolved from thematic analysis [57]. In this approach, multiple coders work independently and subsequently compare their analysis. The degree of consensus reached enables the reliability of the results [57,58]. Initial coding involved creating short summaries from the raw data (eg, “presence therapy is preferred”). Subsequently, the researchers clustered these codes. For example, summaries related to hardware and software were grouped under the theme “technical infrastructure,” while all mentions of AI were sorted to the theme section “AI-Psychotherapy.” In the following team meeting, these themes were merged and organized into a hierarchical category: theme-subtheme system (axial coding). At this stage, 50% (10/20) of the interviews had been coded. With the developed codebook, the 2 independent researchers then coded the remaining half of the interviews (final coding). In the second meeting, the team discussed and finalized the codebook. Final adjustments were made with the assistance of the supervisor or third coder. To underline sufficient data-reporting quality, we applied the checklist for COREQ (Consolidated Criteria for Reporting Qualitative Research) by Tong et al [59]. The checklist is provided in Checklist 1.

## Results

### Overview

Participants’ ages ranged from 20 years to 54 years (mean 25.95, SD 6.91 years). Of the 20 participants, 16 (80%) were identified as female, while 4 (20%) were identified as male. Half of the interviewees (10/20, 50%) reported approximately 3 to 6 hours of digital media use per day. Within the context of their study program, 70% (14/20) had some psychotherapeutic experience and 45% (9/20) reported personal experiences in psychotherapy as patients. Most frequently used personal digital devices were smartphones (20/20, 100%) and laptops (14/20, 70%), followed by tablets (12/20, 60%). Digital media was mentioned for various purposes, primarily for participation in social media (17/20, 85%) and entertainment (16/20, 80%). The use of digital health applications ranged from daily (7/20, 35%) to none (6/20, 30%). In this context, digital support was predominantly used for health tracking purposes (12/20, 60%) or in connection with health insurance applications (10/20, 50%). Further information is presented in Table 1.

**Table 1.** Sociodemographic data of the participants (N=20).

Characteristics	Values, n (%)
Age (y)	
20‐25	14 (70)
26‐30	3 (15)
>30	3 (15)
Study subject	
Psychology	10 (50)
(Human) medicine	10 (50)
Daily media use (h)	
1‐3	6 (30)
3‐6	10 (50)
>6	4 (20)
Experiences with psychotherapy	
Voluntary social year	1 (5)
During study program	14 (70)
Personal experience as a patient	9 (45)

Furthermore, we obtained data regarding the individual use of digital devices in general and for health-related purposes. The data are provided in Table S2 in Multimedia Appendix 2. The results indicate a marked usage behavior and high digital literacy.

### Codebook

**Figure 2.**
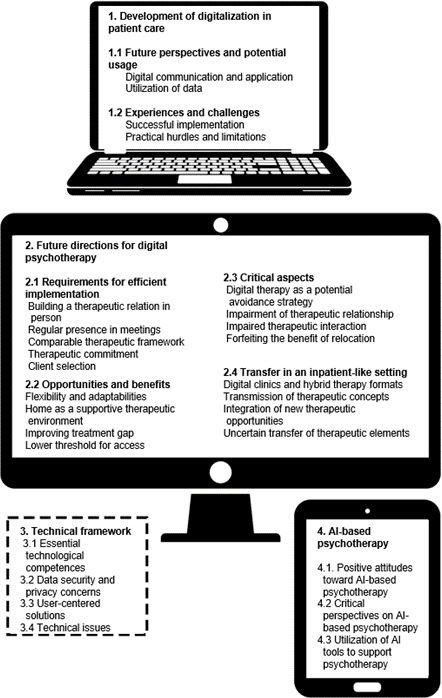
Elaborated codebook with categories, themes, and subthemes. The categories are displayed in separate boxes, where solid lines indicate independent sections and dashed lines represent technical aspects that affect the other themes. AI: artificial intelligence.

The coding process resulted in the emergence of 4 overarching categories: (1) evolution of digitalization in medical practice, (2) future directions for digital psychotherapy, (3) technical framework, and (4) AI-based psychotherapy. A comprehensive overview of the finalized codebook is provided in Figure 2. For transparency and to support clear thematic interpretation, all participant quotations referenced in the results are included in Table S4 in Multimedia Appendix 3.

### Category 1: Development of Digitalization in Patient Care

#### Theme 1.1: Future Perspectives and Potential Usage

This theme included ideas for future digitalization options in somatic medicine. Half of the codes contained a desire to expand existing digital infrastructures, such as providing digital consultation with general practitioners (7/18, 39%) or to make an appointment online (2/18, 11%). In addition, the management and accessibility of digital data emerged as a central concern. Half of the interviewees emphasized the need for the broad implementation of digital medical records, allowing for easier access to patient data across practitioners, involved specialties, or the patients themselves (see quotes 1-3 in Table S4 in Multimedia Appendix 3). Nineteen of 20 (95%) participants generated ideas of digitalization in a medical context, which indicates a general openness to digital health opportunities.

#### Theme 1.2: Experiences and Challenges

A clear positive example for successful digitalization in the analyzed subgroup was the opportunity for digital somatic and psychotherapy treatments (16/60, 27% codes), the use of apps for health purposes (15/60, 25% codes), and digital prescriptions (7/60, 12% codes). However, participants also reported challenges in the digitalization process in different ways; for example, the poor implementation of concepts (see quote 4 in Table S4 in Multimedia Appendix 3) and an insufficient hospital infrastructure (see quote 5 in Table S4 in Multimedia Appendix 3). As Interviewee 17 summarized, “I wish for more digitalization that functions and does not mean more work” (quote 6 in Table S4 in Multimedia Appendix 3). Another concern was the fear of an overreached digitalization, which “should not somehow get out of hand in this way of course, that in the end you do not see the patient in real life at all” (quote 7 in Table S4 in Multimedia Appendix 3), voiced by 25% (5/20) of the participants. Another aspect was that digital interventions could support certain mental diseases in general (quote 8 in Table S4 in Multimedia Appendix 3) or in specific cases, such as tracking food intake while suffering from an eating disorder (quote 9 in Table S4 in Multimedia Appendix 3).

### Category 2: Future Directions for Digital Psychotherapy

#### Theme 2.1: Requirements for Efficient Implementation

The implementation of a digital psychotherapy setting appeared feasible under certain circumstances. Establishing a therapeutic relationship in person prior to initiating digital therapy was a core requirement of the psychotherapeutic process for 15 of 20 (75%) study participants. All participants expressed a preference for some scheduled in-person meetings, rejecting the idea of a fully digital therapy. An aspect highlighted by 14 participants was the need for other framework conditions to remain unchanged. They emphasized the need of working on the same therapeutic contents (quote 10 in Table S4 in Multimedia Appendix 3), in a calm environment (quote 11 in Table S4 in Multimedia Appendix 3), “That the therapy sessions have the same length” (quote 12 in Table S4 in Multimedia Appendix 3), and “there are still emergency addresses (…). That it is clear, that there is always someone available, if it is necessary” (quote 13 in Table S4 in Multimedia Appendix 3). As interviewee 10 expressed, the setting “should not get to be like a service hotline” (quote 14 in Table S4 in Multimedia Appendix 3). Half of the interviewees highlighted the importance of a prior commitment by patients to engage seriously with the digital format, while 7 out of 20 (35%) participants were concerned about a lack of commitment toward the new concept. Another requirement mentioned was the recruitment of suitable patients for digital therapy. Fifty percent (10/20) assumed that suitability depends on individual patient characteristics. Age was considered a decisive factor by 55% (11/20) of the participants, with older patients probably being less suitable than younger patients. In 16 interviews, it was highlighted that the type of the individual mental disease should be considered in the decision whether a digital therapy is suitable. Thirty percent (6/20) of the participants noted that digital therapy might be particularly beneficial for clients with limited physical mobility.

#### Theme 2.2: Opportunities and Benefits

A clear advantage seemed to be the provided flexibility of the setting, mentioned by 85% (17/20). Many anticipated that this flexibility would enable easier integration into everyday life (6/20, 30%), particularly for patients with employment obligations (6/20, 30%). Participants viewed the digital concept as adaptable in situations that would otherwise result in therapy cancellations, such as travel-related difficulties (see quote 15 in Table S4 in Multimedia Appendix 3), “In case of illness, or when you have to look after the children” (see quote 16 in Table S4 in Multimedia Appendix 3) or a lack of access to therapists fluent in a client’s native language (see quote 17 in Table S4 in Multimedia Appendix 3). Another opportunity that was mentioned by 9 interviewees is the possibility of receiving emotional support by receiving therapy from home. In total, 14 interviewees expected that digital concepts could help bridge existing treatment gaps by improving access in underserved regions. Furthermore, 65% (13/20) of interviewees highlighted the lower threshold to access psychotherapy as a clear benefit.

#### Theme 2.3: Critical Aspects

Regarding expected disadvantages, 25% (5/20) of participants alerted that digital therapy might further the possible avoidance of going outside. However, the predominant concern was the expected impairment of interpersonal aspects, mainly concerning the therapeutic alliance between the patient and the therapist, as well as the therapeutic interaction. Seventeen of 20 (85%) respondents believed that digital psychotherapy would impair the therapeutic relationship, making sessions feel less personal (see quote 18 in Table S4 in Multimedia Appendix 3). It seems possible, “that you have concerns or emotions, that only appear, when you are really sitting across from each other” (quote 19 in Table S4 in Multimedia Appendix 3), and it feels better when a therapist is there when you deal with strong upcoming emotions (quote 20 in Table S4 in Multimedia Appendix 3). Participant 15 summarized this by stating “somehow you feel the atmosphere of the person or the chemistry (…). Thus, sometimes I have the feeling, that the screen is more a wall” (quote 21 in Table S4 in Multimedia Appendix 3). Another factor mentioned by 85% (17/20) participants was the reduced insight into nonverbal aspects such as body language, mimic, and gestures (see quote 22 in Table S4 in Multimedia Appendix 3) and the exclusion of aspects of arriving and leaving a therapy room (quote 23 in Table S4 in Multimedia Appendix 3). In addition, the visible image is limited to the upper body (see quote 24 in Table S4 in Multimedia Appendix 3). Sixteen of 20 (80%) interviewees also emphasized that the relocation to the therapist’s office itself provides supporting therapeutic value and would be lost in a digital setting.

#### Theme 2.4: Transfer of Digital Psychotherapy in an Inpatient-Like Setting

The practical transfer of digital psychotherapy to a day-clinic format was imagined by 40% (8/20) of the participants as a fully digital setting, while 30% (6/20) favored hybrid models with varying degrees of digitalization. Three of 20 (15%) interviewees could imagine both formats. Half of the study participants developed ideas for translating existing therapeutic content in digital form. Moreover, across the interviews, new ideas for the digital psychotherapy concept emerged. Most frequently, participants envisioned a mobile application offering supportive additional therapeutic content (11/20, 55%), particularly to create a successful transfer of therapeutic elements into patients’ everyday lives (8/20, 40%), also with regard to the time following discharge from the clinic (see quote 25 in Table S4 in Multimedia Appendix 3). Nevertheless, 40% (8/20) expressed uncertainties or raised open questions regarding the implementation of the digital model.

### Category 3: Technical Framework

#### Theme 3.1: User-Centered Solutions

The subtheme contains aspects that the technical platform for digital therapy should provide. Fourteen of 20 (70%) interviewees imagined therapy through video conferencing. Regarding digital devices, 6 out of 20 (30%) participants underlined the importance of a camera, and 3 out of 20 (15%) participants considered a smartphone to be an inappropriate device for conducting therapy. In 6 interviews, participants described an app as an imaginable platform, and half of them highlighted the importance of a user-friendly design and handling.

#### Theme 3.2: Essential Technological Competences

In 11 interviews, the need for technological competences to gain access to digital psychotherapy was a topic. Nine emphasized patients’ age as an important factor, with older patients probably having more problems with the use of technology, consistent with the concerns about the right client selection. Interviewee 13 described the situation as follows: “I think, you have to be open to the therapy and also have to bring the technical know-how with you. Otherwise, you cannot benefit from it or less, when you are somehow totally overwhelmed with the technology” (quote 26 in Table S4 in Multimedia Appendix 3).

#### Theme 3.3: Data Security and Privacy Concerns

A secure and confidential transmission of medical data, including psychotherapy sessions, was a recurring concern. Forty percent (8/20) pointed out the importance of a technical platform with provided data security. Two of 20 (10%) participants stated that patients may fear being recorded by the therapist. Data privacy concerns also extended to practitioners; accordingly, “vice versa there has to be a protection for the therapist, that there is no software on the other side, which enables a recording of the sessions” (quote 27 in Table S4 in Multimedia Appendix 3). Privacy concerns also arose from the fact that neither participant of an online meeting can see the other person’s entire room (see quotes 28 and 29 in Table S4 in Multimedia Appendix 3). Privacy concerns also appeared in connection with shared patients’ surroundings, “When I think about the shared flat and I do not know if that is the safe space for a profound therapy” (quote 30 in Table S4 in Multimedia Appendix 3).

#### Theme 3.4: Technical Issues

Technical issues emerged in nearly every interview. Seventeen of 20 (85%) interviewees mentioned the importance of a reliable working technological framework. It became clear that various technical disruptions are expected to interfere with the therapy dialogue. The technological side should be “reliably, that the patients can rely on it, that it really takes place every week” (quote 31 in Table S4 in Multimedia Appendix 3). Different concerns showed the vulnerability of a digital therapy session, such as problems with the microphone and internet connection (quote 32 in Table S4 in Multimedia Appendix 3), the quality of the picture of each other (quote 33 in Table S4 in Multimedia Appendix 3), the audio quality (quote 34 in Table S4 in Multimedia Appendix 3), the dependence on a working device (quote 35 in Table S4 in Multimedia Appendix 3), or synchronization problems between audio and video (quote 36 in Table S4 in Multimedia Appendix 3). A more fundamental issue concerned the availability of a working infrastructure. Seven out of 20 (35%) interviewees pointed out that patients might lack the necessary infrastructure (hardware or software); 4 out of 20 (20%) interviewees stated that the clinic might not provide it.

### Category 4: AI-Based Psychotherapy

#### Theme 4.1: Positive Attitudes Toward AI-Based Psychotherapy

It became clear that only a few participants had a positive attitude toward the replacement of a therapist with an AI program. Overall, only 7 codes could be generated over 20 interviews. Expected advantages were a significantly “lower threshold (…), because you can do it 24/7” (quote 37 in Table S4 in Multimedia Appendix 3), with regard to social anxiety, “there is nobody who judges you” (quote 38 in Table S4 in Multimedia Appendix 3), and it seemed to be an option when there is no therapist available locally (quote 39 in Table S4 in Multimedia Appendix 3).

#### Theme 4.2: Critical Perspectives on AI-Based Psychotherapy

A skeptical, if not outright rejecting, attitude was more prevalent. In 80% (16/20) of the interviews, participants emphasized the essential value of human interaction in therapy. The importance of an interpersonal relationship was clearly highlighted (see quotes 40 and 41 in Table S4 in Multimedia Appendix 3). In conclusion, “I would have a problem with accepting AI-generated answers (…) for my requests” (quote 42 in Table S4 in Multimedia Appendix 3). Beyond this, general distrust (7/20, 35%) and concerns emerged that the technical side is not developed enough to enable a sufficient therapeutic intervention.

#### Theme 4.3: Utilization of AI Tools to Support Psychotherapy

In contrast, the use of AI to support making a mental health diagnosis was accepted by 70% (14/20) of the participants. Some even preferred this approach because “you have to be honest, that an AI could maybe reduce something. These two, three sessions diagnostics are never or almost never actually made, especially in the hospital setting, also in the outpatient practice” (quote 43 in Table S4 in Multimedia Appendix 3) and the volume of data could enable an exact diagnosis, the patient could “maybe feel in better hands, because (…) there is a greater data volume to compare” (quote 44 in Table S4 in Multimedia Appendix 3). Furthermore, 75% (15/20) of participants advocated for the use of supportive therapeutic elements like imagination techniques, physical activity, and relaxation instructions or therapeutic diaries.

## Discussion

### Principal Findings

This study explored medical and psychology students’ perspectives on digitalization in medicine and psychotherapy, with a particular focus on our DIPT concept. Participants provided concrete suggestions for supporting digitalization, especially through the expansion of existing structures like teleconsultations, digital appointment scheduling, and digital files. Compared to the existing literature, our findings reflect a more nuanced perspective, containing both enthusiasm and reservation. In recent studies, students’ attitudes appear less focused, claiming a general positive [60,61], while others express an overall skeptical attitude [62]. Positive experiences with telemedicine consultations, as reported by our participants, align with previous literature about patients and providers [63,64]. In a similar setting of integrative medicine during the COVID-19 pandemic, Barth et al [65] reported a particularly good working alliance between the therapists and the patients, which raises hope for the psychotherapeutic sector. Nevertheless, the experience of technical problems was reported repeatedly [63,65], consistent with the concerns expressed in our interviews.

The most extensive thematic focus of this study pertained to students’ opinions toward digital psychotherapy in general and in the context of a possible inpatient setting. We noticed a clear support for the general idea of digital psychotherapy but at the same time a marked hesitation toward a fully digital psychotherapy setting. This rather conservative result was surprising, given the participants’ digital literacy and their frequent use of digital media in general and in health-related contexts. In certain situations—for example, everyday constraints, work-related restrictions, emergency contacts, physical limitations—psychotherapy appears to be accepted; however, fully digital settings seem to be less well-received. Given the assumption that our sample represents a particularly open group with regard to digital treatments, attitudes in the general population may be even more reserved. A closer examination of our study population reveals that our sample consisted of young, predominantly female participants currently enrolled in academic education. This homogeneous study population is consistent with the significantly higher proportion of women in the field of medicine and psychology. The proportion of female students is approximately 70% in medicine [66] and even higher in psychology, at 76.5% [66]. Mackenzie et al [67] reported an association between female gender and a more help-seeking attitude toward mental health treatment, which could consequently lead to a higher response rate of female participants in our study.

Nevertheless, this reported ambivalence toward digital psychotherapy is echoed in current research. In several studies, students show an open attitude toward digital mental health interventions [41,68-70]. Regarding digital psychotherapy, Braun et al [71] reported positive experiences of digital psychotherapy in combination with digital mental health applications. Studies examining students’ opinions of digital psychotherapy remain relatively scarce overall. However, a survey from Gbollie et al [72] revealed that only 12.6% of the students preferred digital solutions over a traditional face-to-face therapy. In another survey that accompanied a group therapy setting, students’ opinions on therapeutic effects were divided [73]. The specific requirements for digital psychotherapy voiced by our participants are consistent with the literature. Building a therapeutic relationship in person prior to digital sessions seems to be favorable [29,74]. Caution regarding changes beyond therapy location seems warranted. Leukhardt et al [74] found that change of location causes a feeling of uncertainty in the psychotherapeutic process. The idea of specific patient suitability seems valid considering studies on factors that influence engagement in digital mental health interventions: older age [75], severe mental health issues [76,77], or even the lack of knowledge of the existence of this type of care seem to be barriers [78]. Conversely, social affiliation [77] or digital literacy [79] facilitate engagement. The stated chances of digital therapy were in line with prior studies. Digital psychotherapy options are valued for their low threshold, greater flexibility, and reduced travel [80]. Nevertheless, important concerns regarding impaired interpersonal and interactional aspects remain significant. Feasibility studies confirm these concerns: patients experienced digital psychotherapy as less personal [29], with more superficial interaction [74]. Frittgen and Haltaufderheide [81] highlight the changes in sensory and embodied aspects of communication. Given both important critical and beneficial aspects, recommendations for a hybrid therapeutic setting, recommended by several of the participants, emerge as the next logical step. The corresponding data seems promising [82], and some even appear better than traditional therapy [83].

Students also identified the technical framework underlying digital care as a central issue. Studies about digital interventions emphasize the importance of easy usability [69,71], likewise a user-centered design [84]. These features are also mentioned by the participants in the present study. Important problems highlighted by the current findings are lack of technical competences or issues with the digital hardware and software infrastructure. These arguments cannot be dismissed and have repeatedly been reported as a relevant implementation hurdle for digital interventions or digital psychotherapy [76,77,83,85]. Another aspect in this theme was awareness regarding the handling of sensitive information, which is reflected in previous research works [69,75,79,84].

Considering the latest developments in digital care, AI was also addressed. In our study, it became clear that AI-guided psychotherapy was largely rejected as a standalone psychotherapeutic agent, whereas AI-guided assistance was widely supported. In recent literature, the use of AI in psychotherapeutic settings appears promising in terms of effectiveness [86-88] and may offer low-threshold access [89]. Critical evidence highlights a lack of emotional depth [90], which aligns with our participants’ skepticism. Kolding et al [87] state that recent studies about AI need to be interpreted with caution because they often lack quality and have limited clinical relevance. At the European level, the AI Act is a regulation that came into force on August 1, 2024 [91], and is intended to protect recipients of AI health services from physical and psychological harm by preventing the approval of applications with potential adverse effects [91]. The Ethics Committee of the Bundesärztekammer has issued a clear statement on this topic: the responsibility for diagnosis, indication, and therapy lies with the physician [92], and only certified treatments should be used [92]. Similarly, the Kassenärztliche Bundesvereinigung emphasizes that AI should be seen as a support tool, not as a replacement for medical services [93].

### Limitations

Despite the insights gained, we still have limitations. The qualitative nature of this interview study can only give results that reflect the outlook and lived experience of the selected study participants. It enables us to understand the investigated themes and develop theories, but the study findings are not generalizable [45]. Nevertheless, we gained valuable knowledge of important factors for mental health treatment and how future mental health professionals want to experience digital psychotherapy, grounded in their life experiences [46]. Although the study group as a whole demonstrated digital literacy and an open-minded attitude toward potential digital psychotherapy treatments, it is noteworthy that only 3 out of 20 (15%) interviewees had received information about digital psychotherapeutic care during their studies, all of whom were psychology students. Given that digital (mental) health treatments are increasingly integrated into routine health care, it is crucial that their application, benefits, and limitations be systematically incorporated into academic curricula at the beginning of professional training. Likewise, Wibberly [94] as well as Kröplin et al [95] argue that the integration of these topics into study curricula is necessary to address the future demands placed on health care providers. In a few cases, study participants were unfamiliar with the concept of a day clinic, and we noticed that this influenced their ability to envision the DIPT concept. Consequently, discussions often shifted toward the aspects of digital outpatient psychotherapy. This context appeared to be more familiar and easier for participants to envision, likely due to their experiences with digital teaching during the COVID-19 pandemic, personal exposure to digital psychotherapy lessons or apps, and contact with digital therapy options addressed during their studies. Furthermore, it is important to keep in mind that researchers, even when following a paradigm of asking open-ended questions without influencing responses, are not free from their own theoretical background and epistemological perspective on the investigated topic [53]. The researchers’ different professional backgrounds allowed for a balanced perspective on both medical and therapeutic topics. First coder and interviewer, a medical doctor (26 years old), was trained in an open-ended interview approach. Students were not directly dependent, neither professionally through work as student assistants, nor in the context of academic performance assessments. Second and third coders were student assistants (study subject was psychology), well trained in qualitative research. Overall, we assume that a pressure-free interview atmosphere was maintained. A logical next step would be a feasibility study of a real-life implementation of DIPT. With the insights gained here, a more successful concept can be developed, expected hurdles can be evaded, and identified opportunities can be realized.

### Conclusion

The results of this interview study led to the conclusion that students are open to more digitalization in health care. Concerning digital psychotherapy, support is more conditional. Hybrid settings were favored that incorporate establishing a therapeutic alliance in person and/or continuing it through in-person meetings during the therapeutic process. A fully AI-guided psychotherapy was rejected, whereas therapeutic supportive tools were welcomed. Technical problems appear to be a relevant hurdle for committing to a digital setting. The obtained aspects can help in implementing adequate digital therapeutic care in a digital inpatient-like or outpatient setting.

## Supplementary material

10.2196/82830 Multimedia Appendix 1 Semistructured interview guide: translated for publication purposes only (originally in German language).

10.2196/82830 Multimedia Appendix 2 Participants’ use of digital media.

10.2196/82830 Multimedia Appendix 3 Quotes for themes and subthemes: translated for publication (originally in German language).

10.2196/82830 Checklist 1 COREQ checklist.

## Abbreviations

AI: artificial intelligence
COREQ: Consolidated Criteria for Reporting Qualitative Research
DIPT: digital inpatient-like psychotherapy
StäB: Stationsäquivalente Behandlung

## References

[1] Mental disorders. World Health Organization.

[2] (2024). NCD-Surveillance-Bericht: Ergebnisse zur Entwicklung Verschiedener Gesundheitsindikatoren in Der Erwachsenen Bevölkerung Bei Hochfrequenter Beobachtung; Stand 2024. https://www.rki.de/DE/Themen/Nichtuebertragbare-Krankheiten/Studien-und-Surveillance/Studien/MHS/NCD-Surveillance-Bericht.pdf?__blob=publicationFile&v=2.

[3] (2019). Krankenkassen Blockieren Sachgerechte Reform Der Bedarfsplanung BPtK: Ländliche Regionen Weiterhin Massiv Benachteiligt. https://api.bptk.de/uploads/20190516_pm_bptk_bedarfsplanung_6d4e1d3d6c.pdf.

[4] (2022). Dokumentation: Wartezeiten Auf Eine Psychotherapie – Studien Und Umfragen [Report in German]. https://www.bundestag.de/resource/blob/916578/53724d526490deea69f736b1fda83e76/WD-9-059-22-pdf-data.pdf.

[5] (2023). Reaktion auf Regierungsangaben: Psychotherapeuten beklagen „schwer erträglichen“ Versorgungszustand [Article in German]. Ärzte Zeitung.

[6] (2025). Fokus: Ambulante Psychotherapie. GKV-Spitzenverband.

[7] Schuld A (2021). Psychosomatik: Argumente für die stationäre Psychotherapie [Article in German]. MMW - Fortschritte der Medizin.

[8] Doering S, Herpertz S, Hofmann T (2023). What kind of patients receive inpatient and day-hospital treatment in departments of psychosomatic medicine and psychotherapy in Germany?. Psychother Psychosom.

[9] Loch AA (2014). Discharged from a mental health admission ward: is it safe to go home? A review on the negative outcomes of psychiatric hospitalization. Psychol Res Behav Manag.

[10] (2021). Report Psychotherapie 2021. 2. Auflage [Report in German]. https://www.dptv.de/fileadmin/Redaktion/Bilder_und_Dokumente/Wissensdatenbank_oeffentlich/Report_Psychotherapie/DPtV_Report_Psychotherapie_2021.pdf.

[11] Stationsäquivalente psychiatrische Behandlung [Article in German]. Deutsche Krankenhaus Gesellschaft.

[12] Bramesfeld A (2023). Die Versorgung von Menschen mit psychischen Erkrankungen in Deutschland aus Perspektive des Gesundheits- und Sozialsystems: Aktuelle Entwicklungsbedarfe [Article in German]. Bundesgesundheitsbl.

[13] Nikolaidis K, Weinmann S, Döring S (2024). Stationsäquivalente Behandlung (StäB) im Vergleich mit vollstationärer Behandlung: 12-Monats-Follow-up Ergebnisse einer mittels Propensity-Score gematchten retrospektiven Kohortenstudie [Article in German]. Psychiatr Prax.

[14] Spannhorst S, Weller S, Thomas C (2020). Stationsäquivalente Behandlung: Eine neue Versorgungsform auch in der Gerontopsychiatrie [Article in German]. Z Gerontol Geriat.

[15] Boege I, Schepker R, Fegert JM (2020). Vom Hometreatment zur stationsäquivalenten Behandlung (StäB): Ein systematischer Review aufsuchender Behandlung in Deutschland [Article in German]. Z Kinder Jugendpsychiatr Psychother.

[16] Schwarz J, Hemmerling J, Kabisch N (2022). Equal access to outreach mental health care? Exploring how the place of residence influences the use of intensive home treatment in a rural catchment area in Germany. BMC Psychiatry.

[17] Rojas G, Martínez V, Martínez P, Franco P, Jiménez-Molina Á (2019). Improving mental health care in developing countries through digital technologies: a mini narrative review of the Chilean case. Front Public Health.

[18] Witteveen AB, Young S, Cuijpers P (2022). Remote mental health care interventions during the COVID-19 pandemic: an umbrella review. Behav Res Ther.

[19] Weitzel EC, Quittschalle J, Welzel FD, Löbner M, Hauth I, Riedel-Heller SG (2021). E-mental-health und digitale Gesundheitsanwendungen in Deutschland [Article in German]. Nervenarzt.

[20] Sifat MS, Tasnim N, Stoebenau K, Green KM (2022). A qualitative exploration of university student perspectives on mindfulness-based stress reduction exercises via smartphone app in Bangladesh. Int J Qual Stud Health Well-being.

[21] Sasseville M, LeBlanc A, Boucher M (2021). Digital health interventions for the management of mental health in people with chronic diseases: a rapid review. BMJ Open.

[22] Lattie EG, Adkins EC, Winquist N, Stiles-Shields C, Wafford QE, Graham AK (2019). Digital mental health interventions for depression, anxiety, and enhancement of psychological well-being among college students: systematic review. J Med Internet Res.

[23] Ferrari M, Allan S, Arnold C (2022). Digital interventions for psychological well-being in university students: systematic review and meta-analysis. J Med Internet Res.

[24] Petrovic M, Gaggioli A (2020). Digital mental health tools for caregivers of older adults-a scoping review. Front Public Health.

[25] Zwerenz R, Becker J, Knickenberg RJ, Siepmann M, Hagen K, Beutel ME (2017). Online self-help as an add-on to inpatient psychotherapy: efficacy of a new blended treatment approach. Psychother Psychosom.

[26] Diel A, Schröter IC, Frewer AL (2024). A systematic review and meta analysis on digital mental health interventions in inpatient settings. NPJ Digit Med.

[27] Gagnon MP, Sasseville M, Leblanc A (2022). Classification of digital mental health interventions: a rapid review and framework proposal. Stud Health Technol Inform.

[28] Fletcher TL, Hogan JB, Keegan F (2018). Recent advances in delivering mental health treatment via video to home. Curr Psychiatry Rep.

[29] Moeller AM, Hansen JP, Andersen PT (2022). Patients’ experiences of home-based psychotherapy via videoconference: a qualitative study. Arch Psychiatr Nurs.

[30] Lamb T, Pachana NA, Dissanayaka N (2019). Update of recent literature on remotely delivered psychotherapy interventions for anxiety and depression. Telemed J E Health.

[31] Markowitz JC, Milrod B, Heckman TG (2021). Psychotherapy at a distance. Am J Psychiatry.

[32] Weightman M (2020). Digital psychotherapy as an effective and timely treatment option for depression and anxiety disorders: implications for rural and remote practice. J Int Med Res.

[33] Smith KA, Blease C, Faurholt-Jepsen M (2023). Digital mental health: challenges and next steps. BMJ Ment Health.

[34] (2024). Hochschulen: Studierende nach Bundesländern [Article in German]. Statistisches Bundesamt (Destatis).

[35] (2024). Digital native. Cambridge Dictionary.

[36] Braun P, Schwientek AK, Angerer P (2023). Investigating information needs and preferences regarding digital mental health services among medical and psychology students in Germany: a qualitative study. Digit Health.

[37] Heesacker M, Perez C, Quinn MS, Benton S (2020). Computer-assisted psychological assessment and psychotherapy for collegians. J Clin Psychol.

[38] Keis O, Grab C, Schneider A, Öchsner W (2017). Online or face-to-face instruction? A qualitative study on the electrocardiogram course at the University of Ulm to examine why students choose a particular format. BMC Med Educ.

[39] Harrer M, Adam SH, Baumeister H (2019). Internet interventions for mental health in university students: a systematic review and meta-analysis. Int J Methods Psychiatr Res.

[40] Harith S, Backhaus I, Mohbin N, Ngo HT, Khoo S (2022). Effectiveness of digital mental health interventions for university students: an umbrella review. PeerJ.

[41] Grüneberg C, Bäuerle A, Karunakaran S (2025). Medical students’ acceptance of tailored e-mental health apps to foster their mental health: cross-sectional study. JMIR Med Educ.

[42] Bhatt S (2025). Digital mental health: role of artificial intelligence in psychotherapy. Ann Neurosci.

[43] Dehbozorgi R, Zangeneh S, Khooshab E (2025). The application of artificial intelligence in the field of mental health: a systematic review. BMC Psychiatry.

[44] Kuper A, Reeves S, Levinson W (2008). An introduction to reading and appraising qualitative research. BMJ.

[45] Bullock A (2016). Conduct one-to-one qualitative interviews for research. Educ Prim Care.

[46] Dicicco-Bloom B, Crabtree BF (2006). The qualitative research interview. Med Educ.

[47] Weller SC, Vickers B, Bernard HR (2018). Open-ended interview questions and saturation. PLoS One.

[48] Hennink MM, Kaiser BN, Marconi VC (2017). Code saturation versus meaning saturation: how many interviews are enough?. Qual Health Res.

[49] (2025). f4 Login – audiotranskription [Website in German]. f4x automatische Transkription.

[50] Rädiker S, Kuckartz U (2020). Focused Analysis of Qualitative Interviews with MAXQDA: Step by Step.

[51] (2025). MAXQDA [Article in German].

[52] Kuckartz U (2018). Qualitative Inhaltsanalyse Methoden, Praxis, Computerunterstützung [Book in German].

[53] Braun V, Clarke V (2006). Using thematic analysis in psychology. Qual Res Psychol.

[54] Braun V, Clarke V (2019). Reflecting on reflexive thematic analysis. Qual Res Sport Exerc Health.

[55] Bennett D, Barrett A, Helmich E (2019). How to…analyse qualitative data in different ways. Clin Teach.

[56] Ryan GW, Bernard HR (2003). Techniques to identify themes. Field Methods.

[57] Braun V, Clarke V (2021). Can I use TA? Should I use TA? Should I not use TA? Comparing reflexive thematic analysis and other pattern-based qualitative analytic approaches. Couns Psychother Res.

[58] Braun V, Clarke V, Hayfield N, Terry G, Liamputtong P (2019). Handbook of Research Methods in Health Social Sciences.

[59] Tong A, Sainsbury P, Craig J (2007). Consolidated criteria for reporting qualitative research (COREQ): a 32-item checklist for interviews and focus groups. Int J Qual Health Care.

[60] Veikkolainen P, Tuovinen T, Jarva E (2023). eHealth competence building for future doctors and nurses - attitudes and capabilities. Int J Med Inform.

[61] Thapa S, Nielsen JB, Aldahmash AM, Qadri FR, Leppin A (2021). Willingness to use digital health tools in patient care among health care professionals and students at a university hospital in Saudi Arabia: quantitative cross-sectional survey. JMIR Med Educ.

[62] Baumgartner M, Sauer C, Blagec K, Dorffner G (2022). Digital health understanding and preparedness of medical students: a cross-sectional study. Med Educ Online.

[63] Barkai G, Gadot M, Amir H, Menashe M, Shvimer-Rothschild L, Zimlichman E (2021). Patient and clinician experience with a rapidly implemented large-scale video consultation program during COVID-19. Int J Qual Health Care.

[64] Garcia-Huidobro D, Rivera S, Valderrama Chang S, Bravo P, Capurro D (2020). System-wide accelerated implementation of telemedicine in response to COVID-19: mixed methods evaluation. J Med Internet Res.

[65] Barth J, Canella C, Oehler M, Witt CM (2021). Digital consultations during COVID-19: a multiperspective mixed-methods study in an integrative medicine setting in Switzerland. J Altern Complement Med.

[66] DatenCHECK 3/25: Was studieren Frauen? Was studieren Männer? – Studierende und Studienanfänger*innen nach Geschlecht [Article in German]. CHE Hochschuldaten.

[67] Mackenzie CS, Gekoski WL, Knox VJ (2006). Age, gender, and the underutilization of mental health services: the influence of help-seeking attitudes. Aging Ment Health.

[68] Topooco N, Fowler LA, Fitzsimmons-Craft EE (2022). Digital interventions to address mental health needs in colleges: perspectives of student stakeholders. Internet Interv.

[69] Dederichs M, Weber J, Pischke CR, Angerer P, Apolinário-Hagen J (2021). Exploring medical students’ views on digital mental health interventions: a qualitative study. Internet Interv.

[70] Van Der Poll R, Coetzee B, Bantjes J (2023). Willing and unwilling digital cyborg assemblages: university students talk about mental health apps. Digit Health.

[71] Braun P, Atik E, Guthardt L, Apolinário-Hagen J, Schückes M (2023). Barriers to and facilitators of a blended cognitive behavioral therapy program for depression and anxiety based on experiences of university students: qualitative interview study. JMIR Form Res.

[72] Gbollie EF, Bantjes J, Jarvis L (2023). Intention to use digital mental health solutions: a cross-sectional survey of university students attitudes and perceptions toward online therapy, mental health apps, and chatbots. Digit Health.

[73] Hunt X, Jivan DC, Naslund JA, Breet E, Bantjes J (2023). South African university students’ experiences of online group cognitive behavioural therapy: implications for delivering digital mental health interventions to young people. Glob Ment Health (Camb).

[74] Leukhardt A, Heider M, Reboly K, Franzen G, Eichenberg C (2021). Videobasierte Behandlungen in der psychodynamischen Psychotherapie in Zeiten der COVID-19-Pandemie: Interviewstudie mit Psychotherapeut*innen und Patient*innen [Article in German]. Psychotherapeut (Berl).

[75] Mayer G, Gronewold N, Alvarez S, Bruns B, Hilbel T, Schultz JH (2019). Acceptance and expectations of medical experts, students, and patients toward electronic mental health apps: cross-sectional quantitative and qualitative survey study. JMIR Ment Health.

[76] Van Assche E, Bonroy B, Mertens M (2022). E-mental health implementation in inpatient care: exploring its potential and future challenges. Front Digit Health.

[77] Borghouts J, Eikey E, Mark G (2021). Barriers to and facilitators of user engagement with digital mental health interventions: systematic review. J Med Internet Res.

[78] Weitzel EC, Schwenke M, Schomerus G (2023). E-mental health in Germany - what is the current use and what are experiences of different types of health care providers for patients with mental illnesses?. Arch Public Health.

[79] Özer Ö, Köksal B, Altinok A (2024). Understanding university students’ attitudes and preferences for internet-based mental health interventions. Internet Interv.

[80] Meier JV, Noel JA, Kaspar K (2023). Understanding psychology students’ perspective on video psychotherapy and their intention to offer it after graduation: a mixed-methods study. Front Psychol.

[81] Frittgen EM, Haltaufderheide J (2022). 'Can you hear me?': communication, relationship and ethics in video-based telepsychiatric consultations. J Med Ethics.

[82] Ehrt-Schäfer Y, Rusmir M, Vetter J, Seifritz E, Müller M, Kleim B (2023). Feasibility, adherence, and effectiveness of blended psychotherapy for severe mental illnesses: scoping review. JMIR Ment Health.

[83] Omylinska-Thurston J, Aithal S, Liverpool S (2024). Digital psychotherapies for adults experiencing depressive symptoms: systematic review and meta-analysis. JMIR Ment Health.

[84] Oti O, Pitt I (2021). Online mental health interventions designed for students in higher education: a user-centered perspective. Internet Interv.

[85] Ramos G, Hernandez-Ramos R, Taylor M, Schueller SM (2024). State of the science: using digital mental health interventions to extend the impact of psychological services. Behav Ther.

[86] He Y, Yang L, Zhu X (2022). Mental health chatbot for young adults with depressive symptoms during the COVID-19 pandemic: single-blind, three-arm randomized controlled trial. J Med Internet Res.

[87] Kolding S, Lundin RM, Hansen L, Østergaard SD (2024). Use of generative artificial intelligence (AI) in psychiatry and mental health care: a systematic review. Acta Neuropsychiatr.

[88] Zhong W, Luo J, Zhang H (2024). The therapeutic effectiveness of artificial intelligence-based chatbots in alleviation of depressive and anxiety symptoms in short-course treatments: a systematic review and meta-analysis. J Affect Disord.

[89] Jurblum M, Selzer R (2025). Potential promises and perils of artificial intelligence in psychotherapy - the AI Psychotherapist (APT). Australas Psychiatry.

[90] Spytska L (2025). The use of artificial intelligence in psychotherapy: development of intelligent therapeutic systems. BMC Psychol.

[91] (2026). Künstliche Intelligenz: AI-Act: Die wichtigsten Fragen zur KI-Verordnung [Article in German]. IHK Region Stuttgart.

[92] Zentrale Ethikkommission (2021). Stellungnahme der Zentralen Kommission zur Wahrung ethischer Grundsätze in der Medizin und ihren Grenzgebieten (Zentrale Ethikkommission) bei der Bundesärztekammer “Entscheidungsunterstützung ärztlicher Tätigkeit durch Künstliche Intelligenz” [Article in German]. Dtsch Arztebl.

[93] (2026). Themenseite: Künstliche Intelligenz [Article in German]. Kassenärztliche Bundesvereinigung (KBV).

[94] Wibberly KH (2024). Preparing mental health providers for the future: the case for moving beyond the elective telehealth course to integrating telehealth training throughout the curriculum. Front Psychol.

[95] Kröplin J, Maier L, Lenz JH, Romeike B (2025). Impact of a “digital health” curriculum on students’ perception about competence and relevance of digital health topics for future professional challenges: prospective pilot study. JMIR Form Res.

