# Supplementary material for: Students’ Perspectives on Digital Psychotherapy—Possible Solutions for Digital Inpatient-Like Care Concepts: Qualitative Interview Study
Source: JMIR Med Educ. 2026 Jun 1;12:e82830. doi: 10.2196/82830 (PMC13225226; doi:10.2196/82830)
Supplement: Multimedia Appendix 1 [file mededu-v12-e82830-s001.docx]

Table S*3:* Semi-structured interview guide: Translated for publication purpose only (originally in German)

Short questionnaire: Sociodemographic data

| 1 | Interview number |  |
| --- | --- | --- |

| 2 | Age |  |
| --- | --- | --- |

| 3 | Gender | male | female | diverse |
| --- | --- | --- | --- | --- |

| 4 | Study subject: | semester: |
| --- | --- | --- |

| 5 | Completed internships/clinical traineeships in the field of psychotherapy? |
| --- | --- |

| 6 | Experience as patient with the field of psychotherapy? | Yes | No |
| --- | --- | --- | --- |

Frequency of use of digital media

| 7 | How often do you use digital media (in h) ?   - 1. daily   2. once a week   3. several times a week   4. several times a month   5. rare   6. never |
| --- | --- |
| 7b | If daily, how many hours approximately? |
| 7c | Do you have a stable internet access? |

| 8 | Which digital media and which digital devices do you use? |
| --- | --- |
| 8b | For what do you use these digital media and digital devices? |

| 9 | Do you use digital media for health matters (e.g. smartwatch, health insurance app etc.) ? | Yes | No |
| --- | --- | --- | --- |

| 9a | If yes, which and how often do you use them? |
| --- | --- |
| 9b | If no, why not ? |

Concept idea: Digital psychotherapy in a day-clinic setting

| 10 | Do you know the concept of “digital day-clinic treatment” or “digital clinic“? | Yes | No |
| --- | --- | --- | --- |

| 10a | No: Could you imagine, what this term means? What comes to your mind? |
| --- | --- |
| 10b | Yes: Could you explain what the term means? |
| To 10: | Core concept of digital day-care setting:  With the ongoing digitalisation in the healthcare sector, new opportunities as well as requirements for patient-centered, area-wide and individual care for people with mental illness are emerging. The innovative concept of a digital clinic should enable patients and practitioners to discover these new chances and use them as best as possible.  The digital clinic offers low-threshold, flexible and multimodal care of different disciplines in a virtual environment. The spatial independence enables unrestricted access, including extern practitioners (experts), and enables an individual treatment intensity.  Digital diagnostics, video consultations and methods of home-monitoring (e.g. in form of wearables and mobile biofeedback) patients have the opportunity to choose flexibly their therapies, integrate them in their everyday life and co-create them more actively. It enables the experience of autonomy and the sense of responsibility for their own therapy.  Evidence-based psychotherapy treatments are delivered via video sessions (a.o. internet-based cognitive behavioural therapy), individual and group treatments that build on another, supplemented by selected psychotherapy apps (e.g. with interactive exercises, videos, audios about psychoeducation, awareness and much more).  All components are integrated in an intuitive-usable/ structured clinic-app, through this virtual interface clients and practitioners come together.  The digital clinic represents a bridge between (day-)clinic and outpatient therapy and enables individual supplementation and development for the holistic treatment of patients.  *In the following we refer to the concept of digital day-clinic treatment as digital psychotherapy |
| 10c | What do you think about this concept? |
| 10d | What do you think about digitalisation in medicine in general? Do you wish for more or less digitalisation in medicine than realized at the moment? |

Qualitative interview

| Experiences and ideas regarding digital mental health care | 1. What are your experiences with digitalisation in medical context? |
| --- | --- |
|  | 1. What are your experiences with digitalisation in psychotherapy? |
|  | 1. What do you think about the realization of digitalisation in medical context? |
|  | 1. Can you envision digital psychotherapy? |
|  | 1. Can you envision digital psychotherapy as a part of a day-clinic setting? |
|  | 1. If yes, what ideas come to mind? |
|  | 1. Which digital media would you propose for it? |
|  | 1. If no, why cannot you imagine digital psychotherapy? |

| Requirements | What is important for you in digital psychotherapy?  Which requirements would be necessary for digital psychotherapy to be comparable to traditional therapy forms? |
| --- | --- |
| Positive aspects | Which advantages of digital psychotherapy in comparison with traditional psychotherapy do you see?  Do you think that some client groups would benefit more than others from digital psychotherapy? |
| Negative aspects | Which disadvantages of digital psychotherapy in comparison with traditional psychotherapy do you see?  Do you think that some client groups would benefit less or would not benefit from digital psychotherapy? |
| Open questions | Do you think that digital psychotherapy can be equally effective like traditional forms of psychotherapy in a clinic or practice? Or do you see differences in efficacy?  If yes, why?  If no, why? |
| Change of perspective | If you would be a therapist, could you imagine offering digital psychotherapy? Which factors would influence your decision?  Are there concerns or expectations?  How important would be the personal contact with clients?  How often should it be?  Why is the personal contact important or less important? Could it be replaced by communication through digital media? |
| Change of perspective II | If you would be a patient, could you imagine making use of digital psychotherapy? Which factors would influence your decision?  Are there concerns or expectations?  How important would be the personal contact to the therapist?  How often should it be?  Why is the personal contact important or less important? Could it be replaced by communication through digital media? |

| Artificial intelligence | Could you imagine trying AI-guided psychotherapy? Are there concerns or expectations?  Are there aspects that would influence your willingness to try it? |
| --- | --- |
|  | Would you try other AI-guided health interventions (e.g. diagnostics of mental disorders, Yoga classes etc.)? |

| Last question | Do you have questions or recommendations for  me? |
| --- | --- |
