# Supplementary material for: Students’ Perspectives on Digital Psychotherapy—Possible Solutions for Digital Inpatient-Like Care Concepts: Qualitative Interview Study
Source: JMIR Med Educ. 2026 Jun 1;12:e82830. doi: 10.2196/82830 (PMC13225226; doi:10.2196/82830)
Supplement: Multimedia Appendix 2 [file mededu-v12-e82830-s002.docx]

*Table S 2*: Participants´ use of digital media

| *Digital devices*  Smartphone  Laptop  Tablet  PC  Console  TV | *User*  20  14  12  4  2  2 | *In* *percent*  (100)  (70)  (60)  (20)  (10)  (10) |
| --- | --- | --- |
| *Function of digital media*  Social Media  Communication  Entertainment  University or/and Work  Information  Relaxation  Inspiration | 17  15  16  14  8  1  2 | (85)  (75)  (80)  (70)  (40)  (5)  (10) |
| *Usage of digital health media*  Daily  Several times a week  Several times a month  1-2x/ month  None | 7  1  1  3  6 | (35)  (5)  (5)  (15)  (30) |
| *Digital health applications*  Health insurance app  Research  Digital appointment booking  Sport  Meditation  Digital Psychotherapy  Digital prescription  Tracking | 10  7  5  2  1  1  5  12 | (50)  (35)  (25)  (20)  (5)  (5)  (25)  (60) |

Note. N=20
