# Supplementary material for: Students’ Perspectives on Digital Psychotherapy—Possible Solutions for Digital Inpatient-Like Care Concepts: Qualitative Interview Study
Source: JMIR Med Educ. 2026 Jun 1;12:e82830. doi: 10.2196/82830 (PMC13225226; doi:10.2196/82830)
Supplement: Multimedia Appendix 3 [file mededu-v12-e82830-s003.docx]

*Table S4*: Quotes for themes and subthemes: Translated for publication (originally in German)

| Subtheme 1.1:  Future perspectives and potential usage  Subtheme 1.2:  Experiences and challenges | [1] “that for example patient data is more easily accessible for practitioners” (participant 1)  [2] “the fundamental idea of a digital patient file is very attractive in my opinion, especially regarding the communication of various medical specialties and for the coordination of a general practitioner” (participant 4)  [3] “when […] a health insurance card, when it contains all data or when you could get somehow access on your own more easily” (participant 6)  [4] “I have the feeling, that many ideas are good, but they somehow can only be used insufficiently in practice” (participant 3)  [5] “in the everyday life in hospital […] you gain a bit more negative experiences with digitalisation in my opinion […] all the programs are really slow. The computers are not working properly. They shut down often and the problem is, when the programs are not working […] then nothing is functioning any more” (participant 4)  [6] “I wish for more digitalisation that functions and does not mean more work” (participant 17)  [7] “should not somehow get out of hand in this way of course, that in the end you do not see the patient in real life at all” (participant 12)  [8] “It is a question, to what extent these systems support the mental illness” (participant 16)  [9] “today I do not track my food any more […] I am a special case, because I had an eating disorder” (participant 12) |
| --- | --- |
| Subtheme 2.1:  Requirements for efficient implementation  Subtheme 2.2:  Opportunities and benefits  Subtheme 2.3  Critical aspects  Subtheme 2.4  Transfer of digital psychotherapy in daycare setting | [10] “everything that you can do in therapy in person should also be realized well digitally” (participant 13)  [11] “That the therapist is still sitting in a calm environment […]. Then maybe from the patient´s side, that he has the same conditions” (participant 9)  [12] “That the therapy sessions have the same length” (participant 15)  [13] “there are still emergency addresses […]. That it is clear, that there is always someone available, if it is necessary.” (participant 19)  [14] “(the setting) should not get to be like a service hotline” (participant 10)  [15] “when patients for example are not […] capable of traveling to the clinic, because the local public transport goes on strike” (participant 1)  [16] “In case of illness, or when you have to look after the children” (participant 11)  [17] “maybe a native speaking psychotherapist” (participant 15)  [18] “that it is not quite that personal and maybe harder to build trust or to show empathy” (participant 9)  [19] “that you have concerns or emotions, that only appear, when you are really sitting across from each other” (participant 9)  [20] “sometimes it feels better, when you sit with the therapist in a room. […] maybe when you have to cry or something, then you do not sit there alone” (participant 12)  [21] “somehow you feel the atmosphere of the person or the chemistry […]. Thus, sometimes I have the feeling, that the screen is more a wall” (participant 15)  [22] “it is part of therapy, somehow see body language, mimic and gestures” (participant 2)  [23] “Appearance, gait, how someone comes into the room? Of course, all of this is cancelled, because we only start the therapy at the moment when we turn on the screen” (participant 7)  [24] “you also need perfect conditions […]. You have to see the face perfectly and the mimic. And posture and things like that, I think it is a bit hard to see when you see someone to the chest” (participant 14)  [25] “to create the transfer on your own. Because you will not be a patient forever and that you have some kind of bridge, how you take things from therapy and apply them alone” (participant 10) |
| Subtheme 3.2  Essential technological competences  Subtheme 3.3  Data security and privacy concerns  Subtheme 3.4  Technical issues | [26] “I think, you have to be open to the therapy and also have to bring the technical know-how with you. Otherwise, you cannot benefit from it or less, when you are somehow totally overwhelmed with the technology.” (participant 13)  [27] “vice versa there has to be a protection for the therapist, that there is no software on the other side, which enables a recording of the sessions” (participant 7)  [28] “And maybe patient with fears of trust or something, because you cannot control everything. You do not see the whole room” (participant 9)  [29] “you will never see the whole room” (participant 11)  [30] “When I think about the shared flat and I do not know if that is the safe space for a profound therapy” (participant 13)  [31] “(the technological side should be) reliably, that the patients can rely on it, that it really takes place every week.” (participant 19)  [32] “the microphone should not rush and the internet connection should be good and it should, if possible, the whole technical side cause problems as little as possible” (participant 3)  [33] “when the picture is pixelated” (participant 8)  [34] “tone […] should be sensitive” (participant 9)  [35] “internet is shutting down completely, device is broken and the course therapy suffers” (participant 14)  [36] “For example time delays in a video conference, distortion between words spoken, mimic and posture” (participant 16) |
| Subtheme 4.1  Positive attitudes towards AI-based psychotherapy  Subtheme 4.2  Critical perspectives on AI-based psychotherapy  Subtheme 4.3  Utilization of AI-tools to support psychotherapy | [37] “ lower threshold […], because you can do it 24/7” (participant 3)  [38] “there is nobody who judges you” (participant 6)  [39] “when I am searching for years for a therapy place and I am not finding anything, I would consider it” (participant 13)  [40] “I would have reservations, that this could replace an emotional relationship experience” (participant 1)  [41] “But I think, psychotherapy is of all medical specialties the area where the humanity plays an important part” (participant 4)  [42] “I would have a problem with accepting AI-generated answers […] for my requests” (participant 7)  [43] “you have to be honest, that an AI could maybe reduce something. These two, three sessions diagnostics are never or almost never actually made, especially in the dayclinic setting, also in the outpatient practice” (participant 5)  [44] “(I would) maybe feel in better hands, because […] there is a greater data volume to compare” (participant 8) |
