# Supplementary material for: Students’ Perspectives on Digital Psychotherapy—Possible Solutions for Digital Inpatient-Like Care Concepts: Qualitative Interview Study
Source: JMIR Med Educ. 2026 Jun 1;12:e82830. doi: 10.2196/82830 (PMC13225226; doi:10.2196/82830)
Supplement: Checklist 1 [file mededu-v12-e82830-s004.pdf]

Table 5: Consolidated criteria for reporting qualitative research (COREQ)

**Domain 1: Research team and reflexivity**<br>*Personal characteristics*		
I.	Facilitator	Rebekka Robitzsch, Lucy Ann Gresser, Patrick Jonas Wollenberg, Tania Lalgi, Sophie Schulz genannt Menningman, Alexander Bäuerle, Martin Teufel
II.	Credentials	Rebekka Robitzsch, Lucy Ann Gresser, Patrick Jonas Wollenberg, Tania Lalgi (M.Sc.), Sophie Schulz genannt Menningmann (B.Sc.), Anita Robitzsch (MD), Alexander Diel (PHD), Alexander Bäuerle (PHD), Martin Teufel (Prof; MD)
III.	Occupation	**Research associate:** Rebekka Robitzsch, Lucy Ann Gresser, Patrick Jonas Wollenberg, Tania Lalgi, Sophie Schulz genannt Menningmann<br>**Postdoctoral researcher:** Alexander Diel, Anita Robitzsch, Alexander Bäuerle<br>**Full-time professor:** Martin Teufel
IV.	Gender	Female: RR, LG, TL, SSgM, AR<br>Male: PW, AD, AB, MT
V.	Experience and training	RR: PHD candidate, assistant doctor for psychosomatic medicine and psychotherapy<br>LG: PHD candidate<br>PW: PHD candidate<br>SSgM: educational background in psychology (M.Sc.), doctoral researcher<br>TL: educational background in psychology (M.Sc.), doctoral researcher<br>AR: postdoctoral researcher, medical doctor for psychosomatic medicine<br>AD: postdoctoral researcher<br>AB: postdoctoral researcher, psychological psychotherapist, habilitated head of research for psychosomatic medicine<br>MT: full-time professor for psychosomatic medicine and psychotherapy
VI.	Relationship with participants	Recruiting a work setting, Recruiting by contacts of the researcher team, Recruiting by flyer with no relationship in advance
VII.	Relationship established	Relationship established in work setting, otherwise no relationship established
VIII.	Participant knowledge of facilitator	From work, otherwise no knowledge
IX.	Facilitator characteristics	No other characteristics were reported about the facilitators

**Domain 2: Study design**

Theoretical framework	
Methodological orientation and theory	Qualitative thematic analysis by Braun and Clark (2006)
Participant selection	
Sampling	Recruiting from 11/03/24- 31/05/24
Method of approach	Recruiting via flyer and personal introduction
Sample size	*N* = 20 participants
Non-participation	Not applicable
Setting	
Setting of data collection	Premises of LVR University Essen
Presence of non-participants	No
Description of sample	Between 20 and 54 years (*M* = 25,95; *SD* = 6,91), 16 females, 4 males
Data collection	
Interview guide	Provided as supplemental material
Repeat interviews	None
Audio/visual recording	Audio recording
Field notes	No
Duration	18 - 56 minutes
Data saturation	Yes
Transcripts returned	No

**Domain 3: Analysis and findings**	
Number of data coders	3
Description of coding tree	Yes (in the results section)
Derivation of themes	(1) evolution of digitalisation in medical practice<br>(2) future directions for digital psychotherapy<br>(3) technical framework<br>(4) AI-based psychotherapy
Software	MaxQDA 2024
Participant checking	No
Reporting	
Quotations presented	Yes
Data and findings consistent	Yes
Clarity of major themes	Yes
Clarity of minor themes	Yes
